# Using amide proton transfer-weighted MRI to non-invasively differentiate mismatch repair deficient and proficient tumors in endometrioid endometrial adenocarcinoma

**DOI:** 10.1186/s13244-021-01126-y

**Published:** 2021-12-11

**Authors:** Yuan Li, Xinyu Liu, Xiaoqi Wang, Chengyu Lin, Yafei Qi, Bo Chen, Hailong Zhou, Qiaoling Wu, Jing Ren, Jia Zhao, Junjun Yang, Yang Xiang, Yonglan He, Zhengyu Jin, Huadan Xue

**Affiliations:** 1 Department of Obstetrics and Gynecology, Peking Union Medical College Hospital, Peking Union Medical College and Chinese Academy of Medical Sciences, National Clinical Research Center for Obstetric and Gynecologic Diseases, Beijing, People’s Republic of China; 2 Department of Radiology, Peking Union Medical College Hospital, Peking Union Medical College and Chinese Academy of Medical Sciences, Shuai Fu Yuan 1#, Dongcheng Dist., Beijing, 100730 People’s Republic of China; 3 Philips Healthcare China, Beijing, People’s Republic of China; 4 Department of Pathology, Peking Union Medical College Hospital, Peking Union Medical College and Chinese Academy of Medical Sciences, Beijing, People’s Republic of China

**Keywords:** Amide proton transfer-weighted, Magnetic resonance imaging, Endometrioid endometrial adenocarcinoma, Mismatch repair deficient

## Abstract

**Objectives:**

To investigate the utility of three-dimensional (3D) amide proton transfer-weighted (APTw) imaging to differentiate mismatch repair deficient (dMMR) and mismatch repair proficient (pMMR) tumors in endometrioid endometrial adenocarcinoma (EEA).

**Methods:**

Forty-nine patients with EEA underwent T1-weighted imaging, T2-weighted imaging, 3D APTw imaging, and diffusion-weighted imaging at 3 T MRI. Image quality and measurement confidence of APTw images were evaluated on a 5-point Likert scale. APTw and apparent diffusion coefficient (ADC) values were calculated and compared between the dMMR and pMMR groups and among the three EEA histologic grades based on the Federation of Gynecology and Obstetrics (FIGO) grading system criteria. Student’s t-test, analysis of variance with Scheffe post hoc test, and receiver operating characteristic analysis were performed. Statistical significance was set at *p* < 0.05.

**Results:**

Thirty-five EEA patients (9 with dMMR tumors and 26 with pMMR tumors) with good image quality were enrolled in quantitative analysis. APTw values were significantly higher in the dMMR group than in the pMMR group (3.2 ± 0.3% and 2.8 ± 0.5%, respectively; *p* = 0.019). ADC values of the dMMR and pMMR groups were 0.874 ± 0.104 × 10^−3^ mm^2^/s and 0.903 ± 0.100 × 10^−3^ mm^2^/s, respectively. No significant between-group difference was noted (*p* = 0.476). No statistically significant differences were observed in APTw values or ADC values among the three histologic grades (*p* = 0.766 and *p* = 0.295, respectively).

**Conclusions:**

APTw values may be used as potential imaging markers to differentiate dMMR from pMMR tumors in EEA.

## Key points

- 3D turbo spin echo amide proton transfer-weighted (APTw) imaging is feasible for detecting endometrioid endometrial adenocarcinoma (EEA).
- APTw values could differentiate mismatch repair deficiency (dMMR) from mismatch repair proficient (pMMR) tumors in EEA.
- ADC values reveal no significant difference between dMMR and pMMR group.

## Introduction

Endometrial carcinoma (EC) is the seventh most common malignancy worldwide and the only gynecological cancer with a rising incidence and mortality rate [1]. A novel molecular classification that accurately reflects underlying tumor biology and clinical outcomes with potential targeted and immuno-oncology treatment strategies for different subgroups was recently recommended for all patients with EC. Immunohistochemical markers, including mismatch repair (MMR) proteins (MLH1, MSH2, MSH6, and PMS2) serve as key components in this molecular classification [2, 3]. Microsatellite instability-high (MSI-H) constitutes an MMR deficiency (dMMR) phenotype that is present in 20–30% of EC patients and leads to the accumulation of high mutational loads [4, 5]. Tumors without dMMR/MSI-H are considered MMR proficient (pMMR)/microsatellite stable (MSS) [6, 7]. An estimated 3–5% of all EC patients are concomitant with hereditary nonpolyposis colorectal cancer which is also known as Lynch syndrome [8]. dMMR is detected in more than 90% of colonic and endometrial tumors in patients with Lynch syndrome [9]. MMR immunohistochemistry should be performed for the pre-screening of Lynch syndrome in all ECs irrespective of histologic subtype [2].

Magnetic resonance imaging (MRI) plays an essential role in the multidisciplinary management of EC, including characterization and staging of neoplasms, treatment decision-making, and subsequent follow-up [10]. Of note, diffusion weighted imaging (DWI), measuring water molecular mobility, has added considerable value to anatomical imaging and facilitated diagnosis [11]. Bhosale et al. reported the use of intravoxel incoherent motion (IVIM)-derived parameters (ADC values and true diffusion coefficient [*D*_*t*_] values) to determine microsatellite (MSI) status in 12 patients with Federation of Gynecology and Obstetrics (FIGO) stage I EC. Nevertheless, these methods may result in conflicting results or overlap in the measured values [12]. The argument that DWI models lack elements for characterizing biological phenomena and the remaining unresolved parameters derived from complex models present current challenges for application of these approaches in diagnosis [13]. As such, the deployment and evaluation of novel functional MRI techniques that provide new perspectives for analysis of MSI ECs is required.

Amide proton transfer-weighted (APTw) MRI was first introduced by Zhou et al. in 2003 [14]. It is a novel contrast-agent-free MRI technique that addresses the need for endogenous molecular imaging in oncology [15] and has proven valuable for the staging and characterization of tumors [16–18]. As a form of chemical exchange saturation transfer (CEST) imaging, APTw MRI is based on frequent exchange between amide protons in small proteins/peptides and the surrounding water protons, and the transfer of nuclear spin saturation from amide protons to water protons [19] results in water proton signal reduction. Studies have reported that APTw values correlate with cell proliferation and can be used as biomarkers of tumor malignancy [14, 15, 20]. APTw MRI has been predominantly utilized for the central nervous system, and its clinical utility extends to diagnosing tumors of the head and neck, breast, lung, prostate, and rectal cancers [21–26]. The use of two-dimensional (2D) APTw imaging for endometrioid endometrial adenocarcinoma (EEA) was first reported by Yukihisa et al. in 2018. In this seminal report, APTw signal intensities were significantly higher for grade 3 EEA than for grade 1 EEA [27]. Recently, this imaging marker was proven valuable in estimating histological features and risk stratification in early-stage EC [28]. In 2021, Li et al. first reported the feasibility of using three-dimensional (3D) APTw imaging for identifying endometrial adenocarcinoma, that its APTw values were significantly higher than that of benign uterine lesions, and also found APTw values exhibited a moderate positive correlation with Ki-67 proliferation status [29, 30]. The purpose of this study was to investigate the utility of 3D APTw imaging for differentiating between dMMR and pMMR tumors in EEA. This study may provide insight into potential application of APTw imaging in identifying MMR status in EEA.

## Materials and methods

### Study population

This study was approved by the Institutional Review Board and complied with ethical committee standards. Written informed consent was obtained from all participants. The sample size was estimated by considering the difference in APTw values between the dMMR and pMMR groups as the primary outcome. The error was set at 0.05, and the power level was set to 80%. Based on data from the pathology department, a standard deviation of 0.4 was expected and a proportion of 0.3 for the dMMR group was proposed. Therefore, a total sample size of 30 was estimated using the G*Power 3 (version 3.1.9.7) sample size calculation program. A higher number of patients was enrolled in consideration of potential dropouts and poor image quality.

**Fig. 1:**
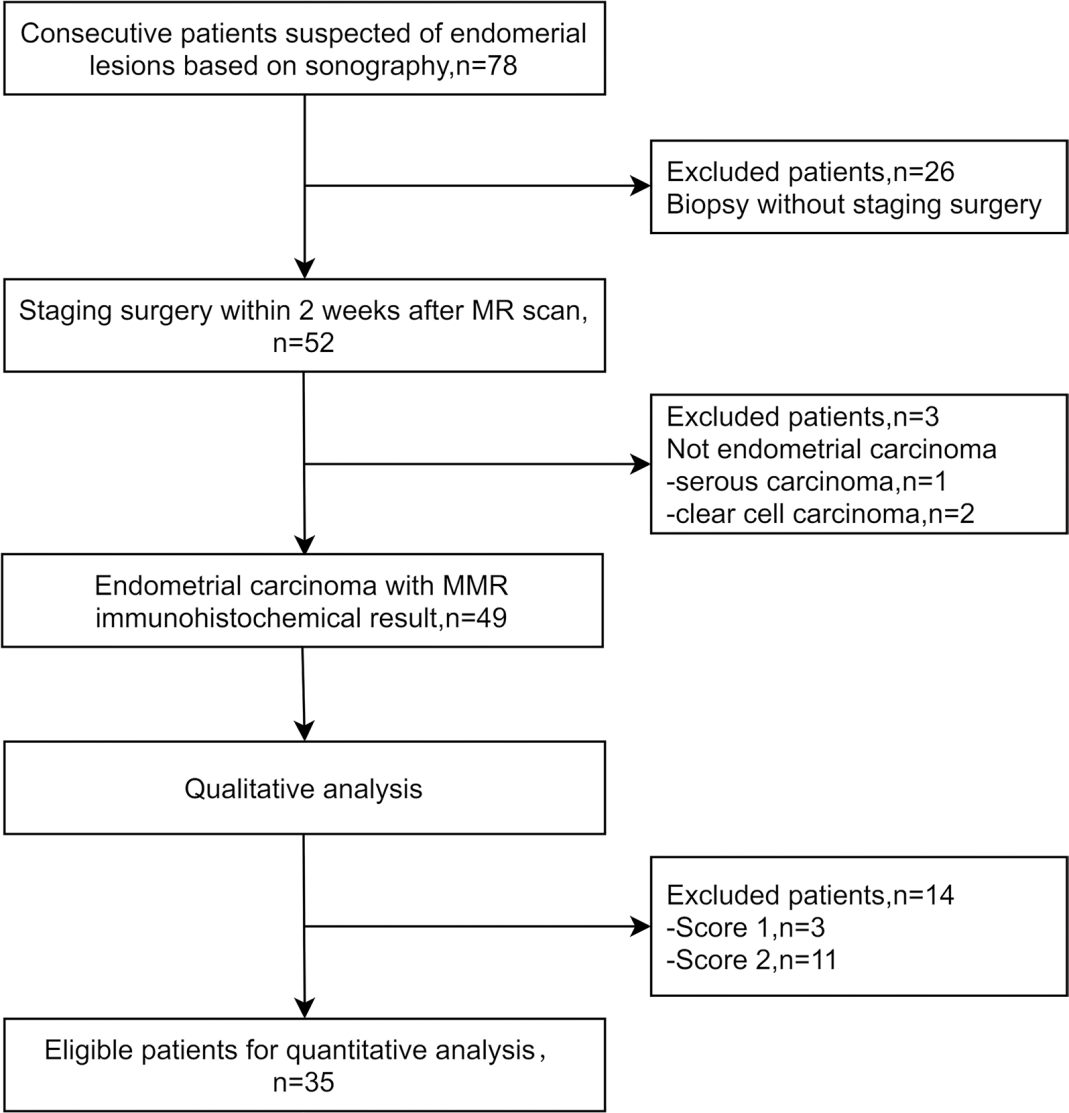
Flow chart of the study cohort. MR = magnetic resonance; MMR = mismatch repair

From January 2018 to October 2020, 78 consecutive female patients aged 25–78 years (mean age, 47.0 ± 14.9 years) with suspicious endometrial lesions and without MR scanning contraindications were prospectively enrolled in this pelvic APTw MRI study. Twenty-six patients without postoperative pathological diagnosis were excluded. Fifty-two patients who underwent staging surgery within 2 weeks after MR scanning were included for further analysis (Fig. 1).

### MRI

An empty bladder was needed. 10 mL of glycerin enema was administered into the rectum 30 min before pelvic MRI examination to reduce air in the rectum and sigmoid. No pre-medications were used to control uterine peristalsis. Pelvic scans were performed with a clinical whole-body 3.0-T MRI unit (Ingenia 3.0 T; Philips Healthcare, Best, the Netherlands). A 16-channel dS Torso coil, which enabled parallel imaging with embedded coils, was applied above the patients. T2-weighted, T1-weighted, and DWI sequences were obtained following with an APTw sequence. A detailed overview of the MRI parameters is listed in Table 1. An ADC map was generated by referring to the signal intensities of DWI with b values of 0 and 1000 s/mm^2^.

**Table 1:** MR imaging parameters details

Parameters	APTw	T1-weighted	T2-weighted	T2-weighted	Diffusion-weighted
Imaging acquisition	3D TSE	TSE	TSE	TSE	EPI
Orientation	Axial	Axial	Axial	Sagittal	Axial
Repetition time/echo time (ms)	7188/5.4	507/8.0	3471/100	3500/100	4656/82
Flip angle (°)	90	90	90	90	90
Field of view (mm^2^)	300 × 243	240 × 300	400 × 400	260 × 260	300 × 218
Matrix (frequency × phase)	120 × 96	320 × 299	400 × 400	512 × 512	100 × 72
Spatial resolution (mm^2^)	2.5 × 2.5	0.75 × 1.0	1.0 × 1.0	0.51 × 0.51	3.0 × 3.0
Slice thickness (mm)	5	4	3	3	4
Slice gap (mm)	0	1	0.3	0.3	1
No. of slices	9	40	41	29	25
ETL	158	4	24	32	…
EPI factor	…	…	…	…	55
SENSE factor	2	1.6	3.5	1.5	1.8
*b* values	…	…	…	…	0,1000
Fat suppression	SPIR	…	…	…	SPAIR
Total image time (min:s)	7:33	3:18	2:36	3:21	1:30

MR = Magnetic resonance; APTw = Amide proton transfer-weighted; TSE = Turbo spin echo; ETL = Echo train length; EPI = Echo planar imaging; SENSE = Sensitivity encoding; SPAIR = Spectral attenuation with inversion recovery; SPIR = Spectral presaturation with inversion recovery. Other key parameters for APTw imaging include 2-s long radiofrequency (RF) pulses at 2-μT power, and 9 acquisitions including 7 different frequency saturation offsets (− 4.3, − 3.5, − 2.7, 2.7, 3.5, 4.3 and − 1560 ppm) and 2 additional acquisitions with echo shifts

A 3D APTw imaging sequence was used in this study, with long radiofrequency (RF) pulses at 2-μT amplitude applied to saturate the amide proton spin signals. In order to generate a continuous 2-s long saturation RF, two RF transmit coils were used and each was turned on for 500-ms alternatively to last four sections. To reduce CEST artifacts from the presence of fat in the pelvis, an asymmetric frequency-modulated pulse (chemical-shift-selective) was applied to suppress fatty tissue MR signals. The APTw sequence was repeated nine times at seven RF saturation offsets (for convenience, the water resonance frequency was set at 0 ppm in the z-spectrum for APT-related offset definitions). Saturation RF pre-pulses were applied selectively at 3.5 ppm which is proven to be the amide proton frequency offset [14]. The APT effect is proportional to the difference of MRI signals with and without saturation pulses at 3.5 ppm. However, the RF magnetic transfer effects have to be compensated for APTw quantification, so the APTw signal is defined as Magnetization Transfer Ratio (MTR) Asymmetry at ± 3.5 ppm. Moreover, B0 inhomogeneity strongly impacts the accuracy because Larmor frequencies of amide protons are shifted if B0 inhomogeneity exists [31]. Hence, two additional acquisitions with different echo shifts of 0.5 ms were performed with the same saturation pre-pulses, and the image volumes with three different echo times (all at offset of + 3.5 ppm) were used to derive the B0 field map. To compensate for B0 inhomogeneity, four more saturation frequency offsets (3.5 ± 0.8 ppm and − 3.5 ± 0.8 ppm) in z-spectrum were measured. The z-spectrum was aligned for each voxel, and the signals at targeting offsets, $S\left( { - 3.5\,{\text{ppm}}} \right)$ and $S\left( { + 3.5\,{\text{ppm}}} \right),$ were calculated using Lagrange interpolation [31]. In summary, APTw values were calculated according to the following equation:

$${\text{APT}}\,{\text{weighting }} = { }\frac{{S\left( { - 3.5\,{\text{ppm}}} \right){ } - { }S\left( { + 3.5\,{\text{ppm}}} \right)}}{{S\left( { - 1560\,{\text{ppm}}} \right)}}$$

where the $S\left( { - 1560{\text{ ppm}}} \right)$ was the signal from one acquisition with saturation RF at extra-large offset (− 1560 ppm) as the control for APTw quantification. The APTw specific absorption ratio (SAR) value was 1.1 W/kg, which fell within the U.S. Food and Drug Administration guidelines. The middle slice of the APTw images was identified based on the largest cross-section of the lesions present on conventional MR images selected by radiologists with 10–17 years of experience in interpreting MR images of the female pelvis.

### APTw image quality analysis

The APTw calculations, including z-spectrum shift and interpolation, were performed online using a MR control console. Raw image datasets were transferred to a workstation (Intellispace Portal; Version 10.1.0.64190; Philips Healthcare, Best, the Netherlands) for post-processing. All MR images were reviewed by three radiologists (Xue HD, He YL, and Lin CY; observers 1, 2, and 3 with 17, 10, and 5 years of experience in interpreting pelvic MR images, respectively), who had previously evaluated over 300 APTw images and were blinded to the patients’ clinical and histopathologic data. Based on the APTw image quality evaluation criteria for uterine cervical cancer [32], the three observers independently ranked the APTw images relative to image quality and measurement confidence on a 5-point Likert scale with respect to image blur, distortion, motion and ghosting artifacts, lesion recognition, and contour delineation. Table 2 summarizes the marking scale and the APTw image scores assessed by the three readers.

**Table 2:** Image quality evaluation of APTw images

Image scores	Scale of marks	Reader 1	Reader 2	Reader 3
5	Good image quality with tumor detectable and lesion contour clearly delineated on APTw images	*n* = 6	*n* = 5	*n* = 6
4	Tumor lesion could be recognized on APTw images, but contour was not so well delineated, reference information on conventional MR images needed for region of interest (ROI) analysis	*n* = 17	*n* = 20	*n* = 18
3	Tumor undetectable without reference to conventional MR images	*n* = 12	*n* = 10	*n* = 11
2	Poor APTw image quality with obvious artifacts, although the tumor lesion was revealed on conventional MR images	*n* = 12	*n* = 11	*n* = 10
1	No lesions were identified on APTw or any conventional MR images	*n* = 2	*n* = 3	*n* = 4

*APTw* amide proton transfer-weighted

### APTw and ADC value measurements

Two observers (Xue HD and He YL; observers 1 and 2) independently measured the APTw values with image quality scores of no less than 3. With reference to conventional MR images, the observers selected a single APTw image and ADC map slice with the maximum lesion area and drew a smooth contour region of interest (ROI) to cover the lesion. The mean APTw and ADC values in areas of the ROIs were recorded.

### Histopathologic and MMR immunohistochemistry analysis

Surgically resected specimens stained with hematoxylin and eosin, and MMR immunohistochemical staining were reviewed by a pathologist (Chen B with 10 years of experience in gynecological pathology), who was blinded to the clinical and imaging data. The aggressiveness of each EEA specimen was categorized into three groups based on the FIGO grading system criteria: grade 1, well-differentiated EEA; grade 2, moderately differentiated EEA; and grade 3, poorly differentiated EEA [33]. The expression of MMR proteins MLH1, MSH2, MSH6, and PMS2 was detected by immunohistochemistry using an FFPE tissue microarray and a Ventana Benchmark XT autostainer (Ventana Medical Systems Inc., Tucson, AZ) according to the manufacturer’s protocols. The absence of nuclear staining in tumor cells was considered a “loss of expression” with intervening stromal positivity serving as an internal control. The complete expression of all four MMR proteins was considered a case of pMMR. The loss of at least one MMR protein was considered a case of dMMR [34].

### Statistical analysis

Statistical analysis was performed using standard statistical software (Prism 8, GraphPad Software, San Diego, CA; SPSS Statistics 23, IBM, NY, USA). For image quality assessment, Kendall’s *W* test was used to evaluate the inter-observer agreement. Inter-class correlation coefficients (ICCs) were computed to evaluate the inter-observer agreement of the APTw value measurements. Kendall’s *W* and ICC values of less than 0.4, 0.41–0.75, and greater than 0.75, were considered to indicate positive but poor, good, and excellent agreement, respectively. The Shapiro–Wilk test was performed to evaluate the normality of the distribution of APTw and ADC values. APTw and ADC values are presented as the mean ± standard deviation. For normally distributed data, Student’s t-test was performed to compare APTw and ADC values between the dMMR and pMMR groups. APTw and ADC values were compared among the three grades using a one-way analysis of variance with Scheffe’s post hoc test. Statistical significance was set at *p* < 0.05. Receiver operating characteristic (ROC) analysis was performed to determine the feasible threshold value with assessment of sensitivity and specificity.

## Results

**Fig. 2:**
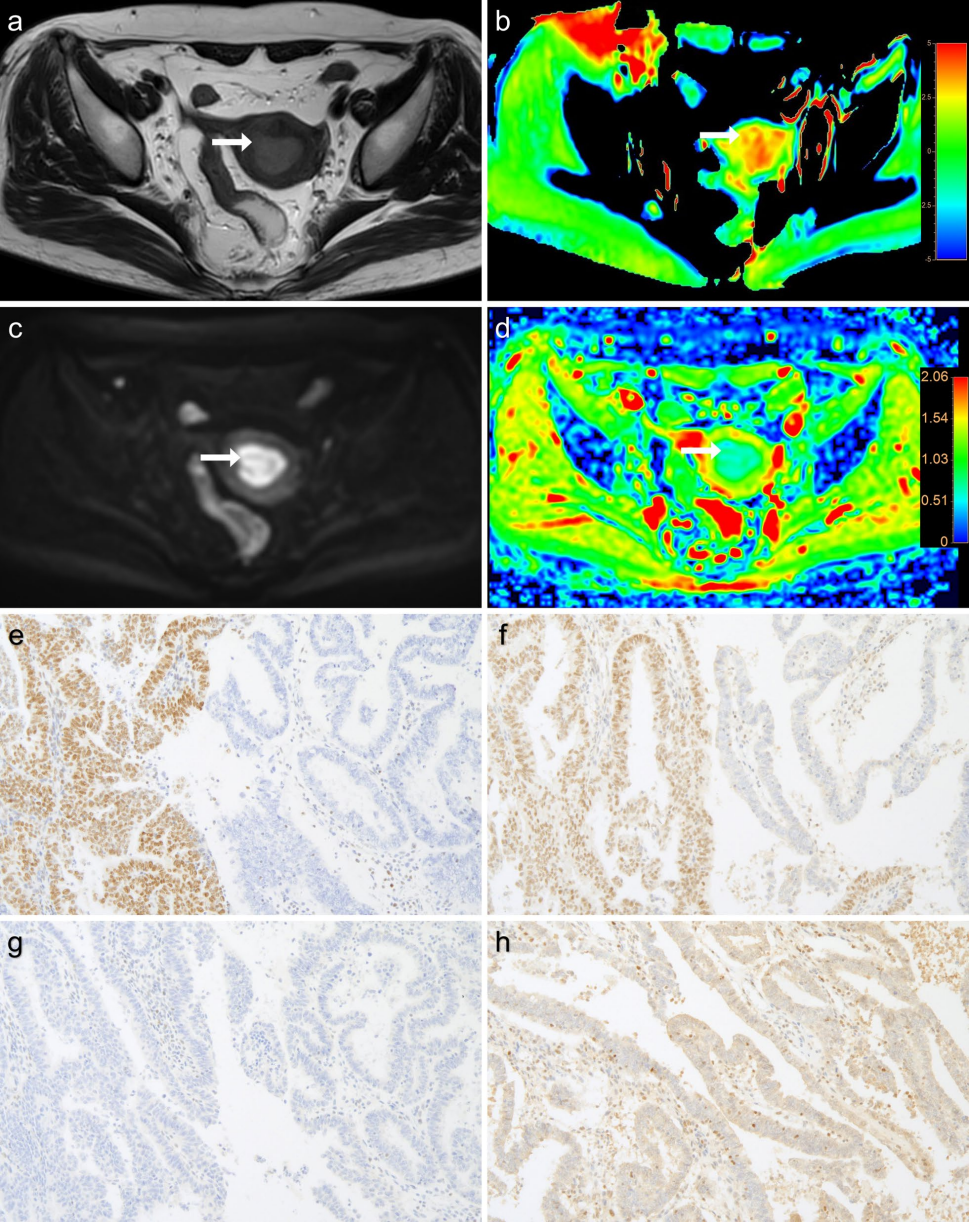
A 56-year-old woman with post-menopause uterus bleeding and mismatch repair deficient (dMMR) endometrioid endometrial adenocarcinoma (EEA), grade 3 with stage IA. MR images of: **a** T2WI; **b** APTw, with mean APTw value 3.0% by two readers; **c** DWI original map (*b* = 1000 s/mm^2^); **d** pseudo colored map of ADC, with mean ADC values 0.721 × 10^−3^ mm^2^/s by two readers. MMR immunohistochemistry analysis revealed the loss of nuclear staining in tumor cells for MLH1 (**e**) and PMS2 (**f**), respectively (×100 magnification); positive staining in stromal cells and normal endometrial glands serves as internal control. Loss of MSH2 (**g**) and MSH6 (**h**) expression, respectively (×100 magnification). Brown stain is positive staining and blue counterstain is indicative of absent or negative staining

**Fig. 3:**
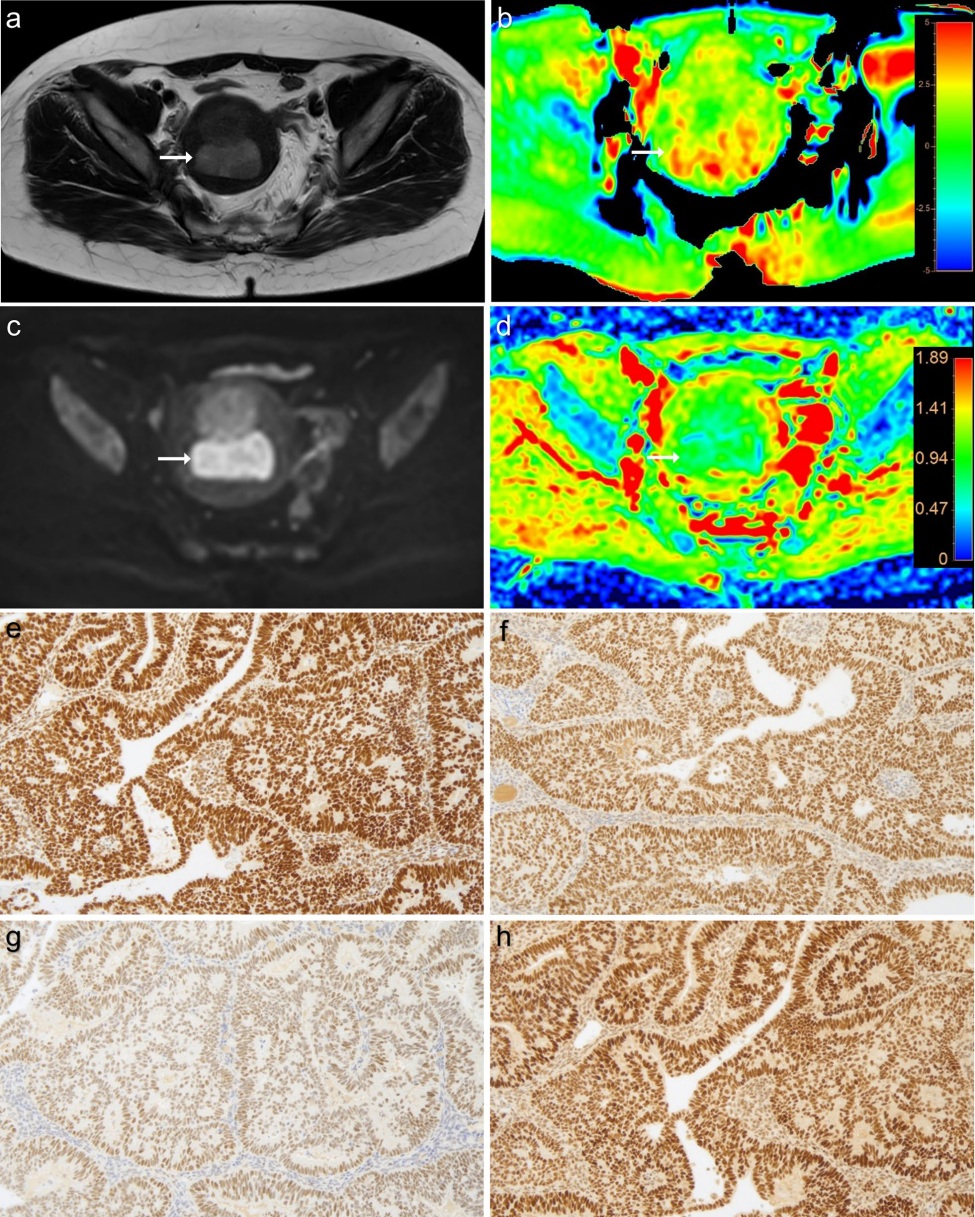
A 53-year-old woman with irregular menstruation and mismatch repair proficient (pMMR) EEA, grade 2 with stage IA. MR images of: (**a**) T2WI; **b** APTw, with mean APTw value 2.8% by two readers; **c** DWI original map (*b* = 1000 s/mm^2^); **d** pseudo colored map of ADC, with mean ADC values 0.763 × 10^−3^ mm^2^/s by two readers. MMR immunohistochemistry analysis (**e**–**h**) revealed the intact nuclear staining for mismatch repair proteins MLH1, PMS2, MSH2 and MSH6 in tumor cells, respectively (×100 magnification)

A flowchart of the study population is presented in Fig. 1. Three patients with other histological types of EC (serous carcinoma, *n* = 1 and clear cell carcinoma, *n* = 2) were excluded. Thus, 49 patients with pathological confirmation of EEA were included in the analysis. MR and immunohistochemical staining images of dMMR and pMMR are presented in Figs. 2 and 3.

### APTw image quality analysis

In total, 49 patients with EEA aged 26–77 years (mean age, 50.9 years) were enrolled for evaluation of APTw image quality. Table 2 summarizes the APTw image scores assessed by the three observers, which exhibited excellent agreement (Kendall’s *W* = 0.867, *p* < 0.001). Most cases were ranked with a score of 4. Poor image quality was observed in 28.6% of the cases due to distortion and artifacts.

### APTw and ADC value measurements

APTw values were obtained in 35 cases of EEA aged 26–76 years (mean age, 50.0 years). Patient demographics are presented in Table 3. The mean ROI area was 315.7 (21.1–1288.0) mm^2^. The APTw values were normally distributed (*p* = 0.890). The mean APTw value was 2.9 ± 0.5% with an inter-observer ICC of 0.985 (95% confidence interval [CI]: 0.971–0.993).

**Table 3:** Characteristics of the 35 patients with EEA

Parameter	No. of patients
Mean age (age range)	50.0 (26–77)
FIGO stage	
IA	29
IB	2
IIIA	1
IIIC2	2
IVB	1
MMR status	
dMMR	9
IA	5
IB	2
IIIC2	1
IVB	1
pMMR	26
IA	24
IIIA	1
IIIC2	1
Histologic grade	
Grade 1	17
Grade 2	15
Grade 3	3

*EEA* endometrioid endometrial adenocarcinoma, *FIGO* Federation of Gynecology and Obstetrics

For ADC value measurements, the mean ROI area of endometrial lesions was 300.2 (32.3–1021) mm^2^. ADC values were normally distributed (*p* = 0.335). The mean ADC value was 0.895 ± 0.101 × 10^−3^ mm^2^/s with an inter-observer ICC of 0.976 (95% CI: 0.954–0.988).

### Comparison of APTw and ADC values between dMMR and pMMR groups

**Fig. 4:**
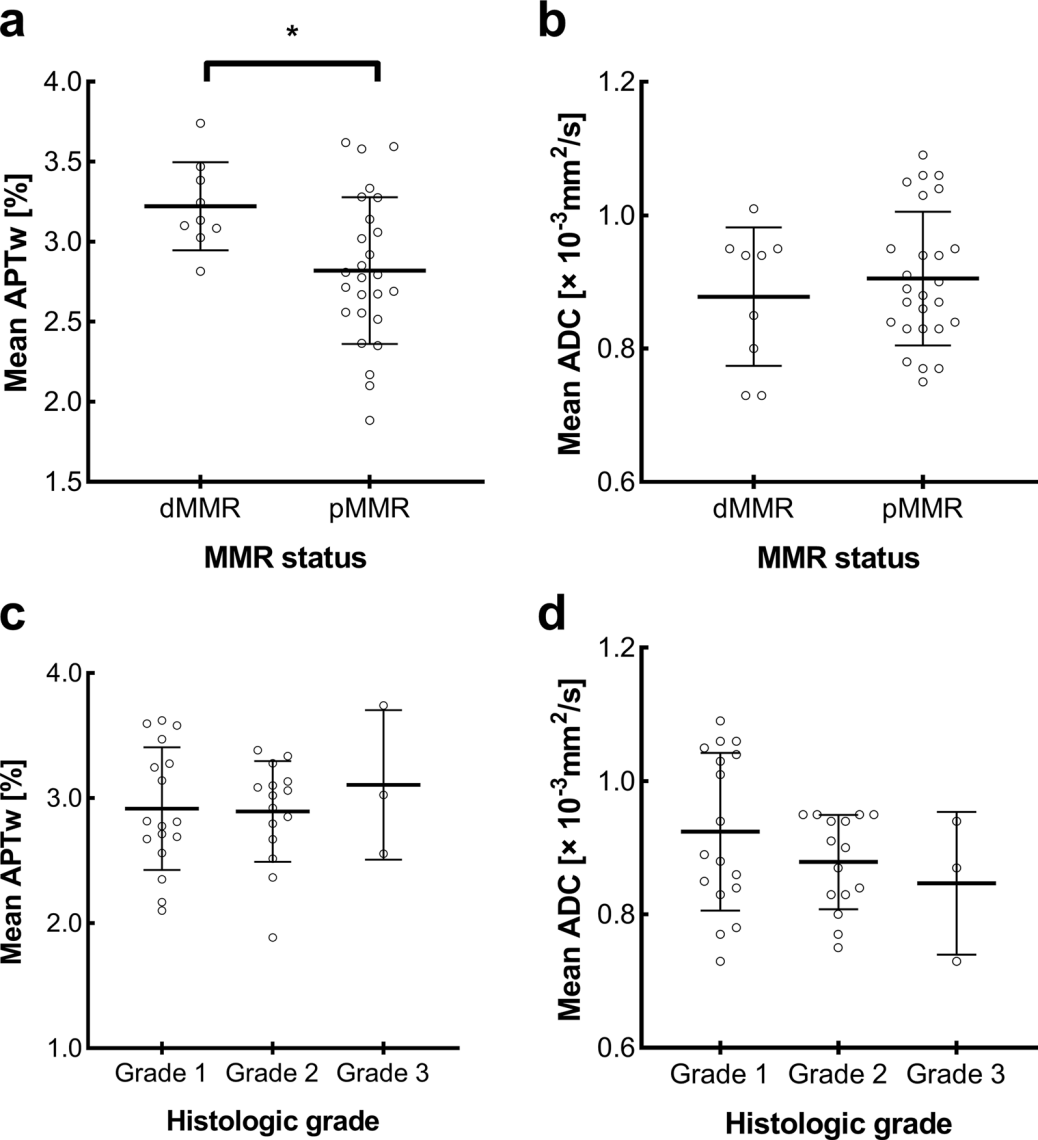
Plots showing individual data points (circles), averages (transverse lines), and standard deviations (vertical lines) of (**a**) mean APTw values and (**b**) mean ADC values of dMMR and pMMR EEAs; **c** mean APTw values and (**d**) mean ADC values of three histologic grades. Individual points were averages of values calculated by two readers. ∗  = Statistically significant difference at *p* < 0.05

**Fig. 5:**
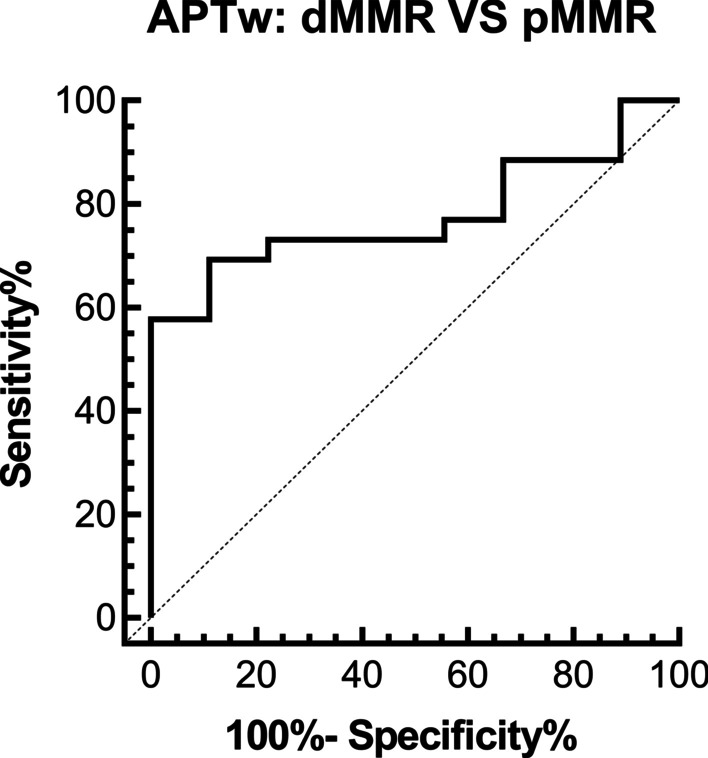
Curves showing mean APTw values by using receiver operating characteristic analysis for differentiating dMMR from pMMR tumors in EEA. The area under the curve (AUC) was 0.778. The feasible threshold values were determined as 3.0% with a sensitivity of 88.9% and specificity of 69.2%

The dMMR and pMMR groups comprised 9 and 26 cases, respectively. The mean APTw value was significantly higher in the dMMR group than in the pMMR group (3.2 ± 0.3% and 2.8 ± 0.5%, respectively; *p* = 0.019; Fig. 4). The area under the curve of ROC analysis for differentiating the dMMR and pMMR groups was 0.778 (Fig. 5). The feasible threshold value was determined to be 3.0%, with a sensitivity of 88.9% and specificity of 69.2%. No significant differences were observed in mean ADC values between the dMMR group (0.874 ± 0.104 × 10^−3^ mm^2^/s) and pMMR group (0.903 ± 0.100 × 10^−3^ mm^2^/s; *p* = 0.476, Fig. 4).

### Comparison of APTw and ADC values between histologic grades

The mean APTw values of grade 1, grade 2, and grade 3 were 2.9 ± 0.5%, 2.9 ± 0.4% and 3.1 ± 0.6%, respectively; the mean ADC values of grade 1, grade 2 and grade 3 were 0.922 ± 0.119 × 10^−3^ mm^2^/s, 0.876 ± 0.070 × 10^−3^ mm^2^/s and 0.845 ± 0.110 × 10^−3^ mm^2^/s, respectively (Fig. 4). No significant differences in APTw or ADC values were observed among the three histologic grades (*p* = 0.766 and *p* = 0.295, respectively).

## Discussion

In this study, we examined the utility of 3D APTw MRI for distinguishing dMMR and pMMR in EEA. Our findings indicate that 3D TSE APTw imaging is a feasible approach for detecting EEA and that APTw values have the potential to differentiate dMMR from pMMR tumors in EEA.

In our cohort, 25.7% (9/35) of patients with EEA presented with dMMR, which was consistent with the literature [35]. The relationship between MSI and prognosis in patients with EC has not been conclusively demonstrated [12]. It is reported that dMMR is a potential biomarker for good responders to PD-L1/PD-1 immunotherapy in EC [36]. MMR immunohistochemistry was recommended to identify MMR status for EC patients in the clinical practice. An invasive endometrial biopsy must be performed for the diagnosis. Nevertheless, it may have 10% false negatives due to the multifocal nature of EC lesions [37] and the specimens obtained are sometimes not sufficient to determine MMR status. As known, MRI plays an essential role in the preoperative evaluation of EC, which is highly specific in the assessment of the depth of myometrial invasion, cervical stromal involvement, and lymph node metastasis [38]. Moreover, MRI must be performed to assess the extension of the disease for patients considering fertility preservation treatments [39]. Multiple imaging techniques are currently being developed to investigate tumor expression of immunotherapy targets PD-L1/PD-1. Based on conventional MR images, dMMR EC tended to be located lower in the uterus (*p* = 0.0366), although most other parameters were not significantly different to those of pMMR EC, including the size (*p* = 0.97), spread (*p* > 0.99), and shape (*p* = 0.76) [40]. Mean ADC values exhibited a trend to be lower in dMMR EC than in pMMR EC, although this finding was not statistically significant (*p* = 0.15) [40], with similar results observed in our study. We demonstrated a correlation between pretreatment APTw values and MMR status. APT technology may enable non-invasive detection of multicellular components of tumor microenvironments to potentially predict response to immunotherapy. Additionally, it might further serve as the non-invasive screening and auxiliary differentiation of EC related to Lynch syndrome. Close surveillance in patients with EC who have dMMR and are subsequently diagnosed with Lynch syndrome is imperative to enable early detection, prevention, and treatment of other cancers [41].

Advanced APTw method was applied in this research, such as 3D acquisition for volumetric imaging, B0 inhomogeneity correction and the long saturation pulses (2-s). Nevertheless, there are some technical challenges. First, APTw value is not necessarily be proportional to cellular proliferation, because it represents not only the concentration of mobile proteins/peptides but also chemical factors such as pH. Secondly, the acquisition resolution of the current APTw imaging method was low; and the strong filtering in post processing resulted in even lower resolution appearance. Thirdly, though B0 field inhomogeneity correction has been applied, field inhomogeneity was still identified as a major challenge in pelvis APTw imaging. Extreme hyper/hypo intensity (> 5% and < − 5%) are presented near thighbone and hipbone, similar to hyperintensity artifacts formed around the skull in brain APTw imaging. We suspect that the Lagrange interpolation algorithm does not work well in the extrapolation conditions with large B0 field deviation. Air in digestive tracts also leads to substantial B0 field distortion, which moves during scanning and causes artifacts that hard to identify, hence administration of glycerin enema was necessary for this examination to reduce air in the rectum and sigmoid. In addition, current APTw application is longer than 5 min. Essentially, it is a 3D TSE sequence with 9 acquisitions; and there is a 2-s saturation pulse within each TR. Less acquisitions reduces acquisition time; however, the resulting image quality is poor. Shorter saturation pulse could also shorten scan duration, but the APT weighting and contrast is reduced. Lastly, we found lower APTw signal in the region closer to the cable of Torso Coil. The coil cable contained residual eddy currents and acted as an unexpected antenna that led to B1 changes during the saturation RF, as a result, it may impact APTw values’ accuracy. Because of these challenges in image artifacts, we spent efforts in evaluating image qualities and exclude APTw images with artifacts in tumor region for further analysis in this study.

There are a few limitations to this study. First, this study was a single-center analysis, which may lead to selection bias. Besides, sample size was small, despite we enrolled adequate patients beyond the required sample size as mentioned above. Future large prospective studies are needed to confirm our findings. Second, although nine APTw image slices were obtained, the APTw values were measured using the single largest area to avoid unnecessary artifacts at the edge of tumors, based on other pelvic APTw imaging studies. Future studies should investigate the APTw values of the whole tumor volume and histograms. Third, the spatial resolution of the APTw imaging was 2.5 × 2.5 mm^2^. As such, some small lesions with tumor areas less than 20 mm^2^ were difficult to evaluate and were excluded from the quantitative analysis. Improvements in the pelvic APTw sequence, such as increasing the spatial resolution, are warranted.

## Conclusion

In conclusion, APTw imaging is a feasible technique for detecting MMR status in EEA. APTw values may be used as potential imaging markers to differentiate dMMR from pMMR tumors in EEA.

## Data Availability

Study data can be made available upon documented request.

## Abbreviations

2D: Two-dimensional
3D: Three-dimensional
ADC: Apparent diffusion coefficient
APTw: Amide proton transfer-weighted
AUC: Area under the curve
CEST: Chemical exchange saturation transfer
dMMR: Mismatch repair deficiency
DWI: Diffusion-weighted imaging
EC: Endometrial carcinoma
EEA: Endometrioid endometrial adenocarcinoma
FIGO: Federation of Gynecology and Obstetrics
ICC: Inter-class correlation coefficient
MMR: Mismatch repair
MRI: Magnetic resonance imaging
MSI: Microsatellite instability
MTR: Magnetization transfer ratio
pMMR: Mismatch repair proficient
RF: Radiofrequency
ROC: Receiver operating characteristic
ROI: Region of interest
SAR: Specific absorption rate
TSE: Turbo spin echo
